# Probing the photophysical properties of fluorescent proteins using photoacoustic pump-probe spectroscopy and imaging

**DOI:** 10.1016/j.pacs.2025.100738

**Published:** 2025-06-06

**Authors:** Farzin Ghane Golmohamadi, Amna Mehmood, Hoang Trong Phan, Franz-Josef Schmitt, Jan Laufer

**Affiliations:** Institut für Physik, Martin-Luther-Universität Halle-Wittenberg, von-Danckelmann-Platz 3, Halle (Saale) 06120, Germany

**Keywords:** Molecular imaging, Photoacoustic imaging, Pump-probe excitation, Fluorescent protein

## Abstract

Pump-probe excitation of fluorophores has been shown to overcome the limitations of conventional multiwavelength imaging and linear unmixing approaches by providing fluorophore-specific contrast whilst eliminating the dominant background signal of endogenous chromophores. In this study, methods for generating pump-probe signals and images are investigated that rely on changing 1) the pump wavelength whilst keeping the probe wavelength fixed, 2) the probe wavelength whilst keeping the pump wavelength fixed, and 3) the time delay between the pump and probe pulse. Time-resolved PA signals were generated in purified solutions of genetically expressed red fluorescent proteins Katushka, mNeptune, and mCardinal in a cuvette. Spectra of the difference signal amplitude were found to correlate with the absorption and emission spectra. The difference signal plotted as a function of time delay also showed characteristic features for each protein. To demonstrate the capability of multiplexed imaging, the spatial distributions of Katushka and mNeptune were recovered from 2D difference images of a phantom. This study demonstrates that methods based on pump-probe excitation can be used to probe the photophysical properties of fluorophores. By detecting changes in these properties due to a stimulant, such as pH, the methods may find application in biosensing of the cellular microenvironment.

## 1 Introduction

Molecular photoacoustic (or optoacoustic) imaging combines strong absorption-based contrast of optical methods with high resolution of ultrasound imaging and has been shown to enable the visualization of contrast agents *in vivo*, such as molecular dyes, nanoparticles, and genetically expressed proteins and pigments [1], [2], [3], [4], [5]. Photoacoustic (PA) images are assumed to show the spatial distribution of the initial pressure, which is often assumed proportional to the local abundance of the chromophores. Conventional methods for recovering relative concentrations typically involve multiwavelength image acquisition and some form of linear spectral unmixing. However, the application of these methods *in vivo* is challenging. Injected or genetically expressed contrast agents tend to accumulate weakly in the target tissue, resulting in low image contrast compared to the dominating background of endogenous chromophores, such as hemoglobin. In addition, the light transport in tissue causes spectral and structural corruption [6], invalidating the assumption of a linear relationship between chromophore concentration and PA image intensity [7]. The challenges of what is known as the optical inverse problem [8] are compounded by image reconstruction artefacts that arise because of the often unavoidable limited view detection apertures [9], [10].

To overcome these limitations, experimental approaches aimed at generating image contrast by exploiting specific photophysical properties of exogenous chromophores are an attractive option. Pump-probe excitation has been shown to overcome some of the limitations of conventional molecular imaging approaches when applied to the detection of fluorescent contrast agents in tomographic images [11], [12], [13], [14], [15]. The method relies on the measurement of a difference signal obtained from two PA signals generated using simultaneous and time-delayed pump and probe pulses (typically a few ns). This technique has been shown to provide an unambiguous and fluorophore-specific contrast mechanism that eliminates the overwhelming background signal. More importantly, pump-probe excitation has the potential to allow the recovery of the photophysical properties of fluorophores. This can be achieved by varying key parameters of photoacoustic excitation, such as the time delay and the wavelengths of the pump and probe pulses. For example, changes in the fluorescence lifetime can be detected in PA images acquired as a function of the pump-probe time delay [16]. The efficiency of the pump-probe contrast mechanism also depends on the wavelength dependence of the absorption and emission cross sections. By varying pump and probe wavelengths, the amplitude of the PA difference signal is expected to correlate, to a first approximation, with the absorption (or excitation) and emission spectra [15] of the fluorophore. It may therefore be possible to extract the optical properties of fluorophores from PA images, which would enable multiplexed imaging or biosensing of the microenvironment if the properties change due to a stimulant, such as pH. A potential application of this method is PA tomography of genetically expressed fluorescent proteins, which are an attractive option because a significant proportion of available proteins absorb in the visible and near-infrared wavelength region where the optical penetration depth is greatest. Genetically expressed reporters, including fluorescent proteins, are usually recovered from PA images using multiwavelength excitation and linear spectral unmixing [17], [18], [19].

In this study, methods are investigated that rely on changing 1) the pump wavelength whilst keeping the probe wavelength fixed, 2) the probe wavelength whilst keeping the pump wavelength fixed, and 3) the time delay between the pump and probe pulse. A dual-OPO laser system provided pump and probe pulses with precisely controlled time delays. The potential application to multiplexed reporter gene imaging is demonstrated in a phantom experiment. The methods for probing the properties of fluorophores using pump-probe excitation, the experimental set-up for measuring time-resolved PA signals and images, the signal processing methods, and the experimental protocols are described in Section 2. The experimental results are presented in Section 3, the discussion is in Section 4, and the conclusions are given in Section 5.

## 2 Materials and methods

### 2.1 Generation of PA pump-probe signals in fluorophores

**Fig. 1:**
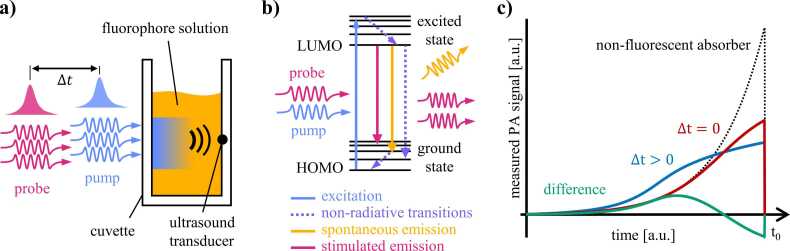
(a) Schematic of a cuvette filled with purified fluorescent protein solution illuminated by pump and probe pulses at a time delay Δt. (b) Simplified Jablonski diagram of electronic transitions under pump-probe excitation. (c) Schematic of the initial compressive part of measured time-resolved PA signals for a time delay ofΔt=0 (red line) and Δt>0 (blue line) from which a difference signal is calculated (green line). A PA signal, measured in a nonfluorescent absorber (black dotted line) with the same absorption coefficient as the fluorophore, using both time delays is shown for comparison.

Pump-probe excitation of PA signals [16], [20] involves the illumination of a fluorophore solution by two excitation pulses separated by time delay Δt as illustrated schematically in Fig. 1a, which shows a simplified measurement geometry for PA spectroscopy. The excitation pulses generate PA waves in a sample solution in a cuvette, and the acoustic transients are detected by an ultrasound transducer. The pump and probe wavelengths coincide with the peaks of the absorption and emission wavelengths of the fluorophore, respectively.

Fig. 1b shows a simplified Jablonski diagram of the electronic transitions during pump and probe excitation. Upon absorption of a pump photon, an electron is elevated from the highest occupied molecular orbital (HOMO) to a higher energy level from where it relaxes to the lowest unoccupied molecular orbital (LUMO) via non-radiative transition, i.e., by converting optical energy to heat. The electron may return to the ground state spontaneously via non-radiative relaxation or by emitting a fluorescence photon. Fluorophores can remain in an excited state for several nanoseconds, which is comparable to the duration of typical excitation pulses used in PA imaging. In addition to the loss of energy to fluorescence, long lifetimes lead to fewer excitation-deexcitation cycles per pulse compared to absorbers with fast transitions and give rise to ground state depopulation, which manifests itself as a transient reduction in absorption at the pump wavelength. As a result, the distribution of thermalized energy, and hence initial pressure, differs from that generated in chromophores with predominately fast (ps) non-radiative transitions. The sign and magnitude of the difference depend on the local photon density and fluorophore concentration [20]. Fig. 1c illustrates the effect of the excited state lifetime on the time-course of PA signals. In non-fluorescent absorbers, the exponential decay of the fluence follows the Beer-Lambert law and is encoded on to the compressive part of the signal (black dashed line) [21]. If fluorophores are excited by single pump pulses (or pump and probe pulses separated by a sufficiently long time delay), ground state depopulation causes lower signal amplitudes in regions of high fluence, e.g., adjacent to the illuminated cuvette window (blue line in Fig. 1c, Δ*t* > 0), and affects the wavelength dependence of the PA signal amplitude which often deviates from reference absorption spectra [22], [23]. The reduced absorption in the superficial layers also allows the light to penetrate deeper into the solution, increasing the fluence (and initial pressure) at greater depths. By illuminating the sample with simultaneous pump and probe pulses (Δt = 0), long-lived excited states are depleted. The probe pulse causes stimulated emission at femtosecond time scales, which increases the population of molecules in the ground state and therefore the number of pump photons that are absorbed within the same pulse. The additional excitation-relaxation cycles change the thermalized energy, and therefore the PA signal amplitude (Fig. 1c, red line).

To generate fluorophore-specific contrast, two measurements are made. First, a PA signal is generated using pump and probe pulses at a time delay longer than the fluorescence lifetime, τ (blue line, Fig. 1c). Under these conditions (and assuming insignificant absorption of probe photons), the effect of the probe pulse on thermalized energy is negligible since most molecules will have returned to the ground state. The PA signal is then almost identical to that generated using single (pump) pulse excitation. Second, PA waves are excited using simultaneous pump and probe pulses (Δt=0, dark red line in Fig. 1c), and a difference signal is obtained by subtracting the two measurements (green line, Fig. 1c). Since the conditions of thermal and stress confinement are met in both cases if Δt is on the order of few ns [24], this contrast mechanism is not observed in non-fluorescent absorbers, and the contribution of endogenous tissue chromophores to difference signals is zero. Other electronic transitions, such as two-photon absorption, excited state absorption, and intersystem crossing, are considered to have a negligible effect at the fluences used in this study.

### 2.2 Multiwavelength and time-delayed pump-probe excitation

**Fig. 2:**
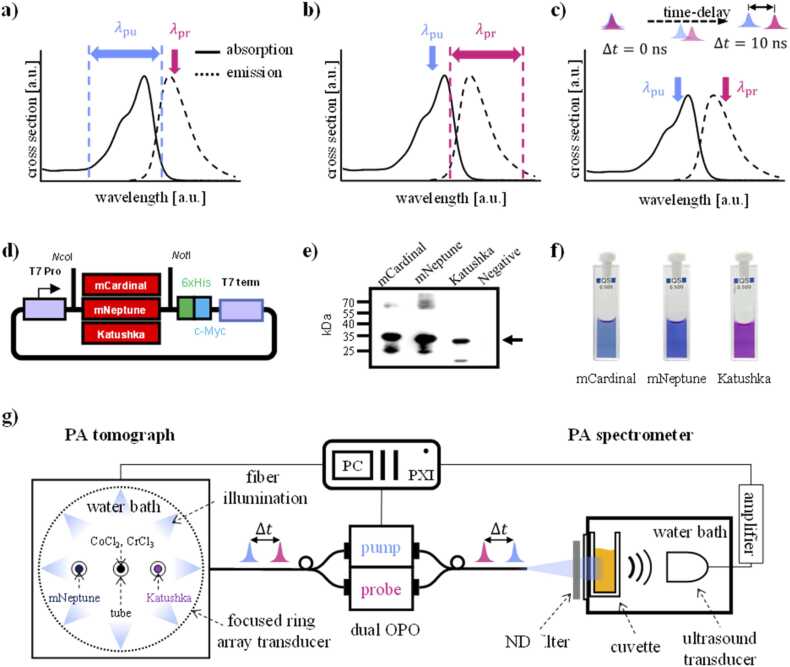
Methods for pump-probe excitation, preparation of the protein samples, and the experimental set-ups for PA spectroscopy and imaging. (a-c) Schematic representation of three methods for pump-probe excitation, which relied on a) a fixed probe wavelength (*λ*_pr_) and scanned pump wavelength (*λ*_pu_), b) a fixed *λ*_pu_ and scanned *λ*_pr_, and c) varying the time delay between pump and probe pulses. d) Expression cassettes for bacterial expression of mCardinal, mNeptune, and Katushka. T7 Pro: T7 promoter; 6xHis: 6 histidine residues; c-Myc: c-Myc tag; T7 term: T7 terminator. e) Expression confirmation of mCardinal, mNeptune, and Katushka using Western blot. 15 µg of total soluble protein was loaded in SDS-PAGE. The arrow indicates the positions of mCardinal, mNeptune, and Katushka. Negative: proteins were extracted from BL21 cells without recombinant expression vectors. f) Photograph of purified protein solutions, from left to right mCardinal, mNeptune, and Katushka in PBS. g) Schematic of the experimental setup for cuvette-based pump-probe spectroscopy and PA tomography of a phantom.

In this work, three methods for pump-probe excitation of fluorophores are evaluated as illustrated in Fig. 2a-c. Fig. 2a shows the first method, which will be referred to as pump wavelength scan in this paper. It involves the acquisition of difference signals as a function of the pump wavelength while the probe wavelength remains fixed. The pump wavelength range corresponds to the absorption spectrum while the probe wavelength is kept at or near the maximum of the emission spectrum. The wavelength dependence of the difference signal, i.e., the change in initial pressure, is therefore expected to resemble that of the absorption spectrum. The second method is illustrated in Fig. 2b and will be referred to as probe wavelength scan. It relies on tuning the probe wavelength across the emission spectrum of the fluorophore while the pump wavelength is kept constant near the absorption maximum of the fluorophore. Since the wavelength-dependence of the efficiency of stimulated emission is considered proportional to the spectrum of the emission cross section [20], the wavelength dependence of the difference signal may be expected to be in qualitative agreement with the fluorescence emission spectrum. Fig. 2c illustrates the third method, which is based on acquiring difference signals as a function of the time delay between pump and probe pulses. The difference signal amplitude is at a maximum when the time delay between pump and probe pulses is minimal. Since the population of proteins in an excited state decreases with time, the difference signal amplitude diminishes with increasing time delay as we have shown previously using a mechanical delay line [16]. In this work, a dual-OPO laser system allowed the time delay to be adjusted electronically from 0 to 1 ms in increments of 1 ns.

### 2.3 Protein samples

Bacterial expression vectors for red fluorescent proteins Katushka, mNeptune, and mCardinal were created (Fig. 2d) and expressed in *E. coli*. The proteins were extracted and verified using Western blot (Fig. 2e) and purified using immobilized metal affinity chromatography. The proteins were dialyzed against phosphate-buffered saline (PBS) to remove unwanted contaminants and salts from the solution. Purified protein solutions (Fig. 2 f) were concentrated using centrifugation, separated in SDS-PAGE, and stained with Coomassie blue for purity analysis using ImageJ densitometry, and stored at −18°C. The purity of the protein solutions was > 90 %. A UV–visible spectrophotometer (ThermoFisher Scientific) and a photoluminescence spectrometer (Agilent Technologies) were used to measure absorption and emission spectra, respectively. A time-resolved fluorescence spectroscopy technique was employed to determine the fluorescence lifetimes [16], which were 1.4 ns for mNeptune and mCardinal, and 2.0 ns for Katushka. To prepare the samples, a specific volume of the concentrated protein solution was removed from the stock and diluted with PBS buffer (pH 7.4). The concentrations of the samples used for pump-probe measurements were 50 µM for mCardinal, 65 µM for mNeptune, and 67 µM for Katushka, and were calculated from measured absorption spectra and the respective molar extinction coefficients [25], [26].

### 2.4 Experimental setup

Fig. 2 g shows a schematic of the experimental setup for PA spectroscopy and imaging. A dual OPO laser system (Innolas Laser GmbH, Germany) generated pump and probe pulses with a duration of 3 ns (FWHM) in the wavelength range of 420–680 nm at a repetition rate of 100 Hz. The output of the dual-OPO system was coupled into a bifurcated fiber bundle (CeramOptec, Germany) to guide the excitation pulses to a PA spectroscopy setup and a PA tomograph. The fiber bundle also produced axially co-aligned pump and probe beams at its distal end. The unattenuated light fluence at the cuvette, $\Phi_{0}$, varied from 8 mJ/cm² to 16 mJ/cm² across the pump wavelength range, and 8 mJ/cm² to 19 mJ/cm² across the probe wavelength range. The excitation pulses were guided to the tomograph using a 1-to-8 fiber bundle.

PA spectroscopy measurements involved generating PA waves in a custom-made cuvette filled with purified protein solutions. The cuvette had a pathlength of around 5.3 mm and a diameter of 18.0 mm to accommodate the typically small sample volumes. The cuvette was held inside a water bath at room temperature (22 °C). PA waves were detected using a matched-load planar PVDF transducer (19 mm dia.) with a bandwidth of 20 MHz at −6 dB and a cutoff frequency of 37 MHz (Precision Acoustics), amplified using a 20 dB voltage amplifier (Femto Messtechnik GmbH). Time-resolved PA signals were acquired with a digitizer card (National Instruments). The setup was calibrated with respect to pulse energy using an energy meter (Ophir Photonics). Multiwavelength PA signals were acquired using pump-probe excitation and conventional single pulse excitation at three fluences by attenuating the output of the OPO lasers using neutral density filters (Comar Instruments), i.e., $\Phi_{0}$, $\Phi_{0}/2$, and $\Phi_{0}/4$. The transmittance spectra of the ND filters were measured using a UV-Vis spectrometer (Thermo Fisher Scientific). PA signals were measured at pump-probe time delays ranging from 0 ns to 10 ns in increments of 1 ns. To compare the data obtained from pump-probe measurements with those of conventional methods, PA signals were also acquired using multiwavelength single-pulse excitation.

A custom-made PA tomography system (PhotoSound Technologies) was employed to acquire 2D multiwavelength pump-probe images of a phantom, which consisted of three translucent silicone tubes (Silastic, 1.47 mm inner dia., 1.96 mm outer dia.) filled with Katushka, mNeptune, and a non-fluorescent solution of CrCl_3_ and CoCl₂. The phantom was immersed in a water bath at room temperature (22°C). The absorption coefficient of the CrCl_3_/CoCl₂ solution ranged from 1 cm^−1^ to 4 cm^−1^ at the pump and probe wavelengths used in this study (Fig. S1, Supplementary Information). PA signals were measured by the tomography system, which is equipped with a focused ring array ultrasound transducer (512 elements, Imasonic, 5.5 MHz center frequency, bandwidth 55 %), and recorded with custom software (LabVIEW). Pump-probe images of a phantom were captured during a pump wavelength scan from 500 nm to 640 nm at 10 nm increments. The pump-probe time delay was 10 ns and signal averaging was used (N = 10).

### 2.5 Signal and image processing

The measured time-resolved signals, $S_{m}\left(t\right)$, were normalized with respect to the pump and probe pulse energies using,

(1) $$S\left(t\right)=k\;S_{m}\left(t\right)\mathrm{with}k=\frac{\mu_{a}\left(\lambda_{\mathrm{pu}}\right)\;+\;\mu_{a}\left(\lambda_{\mathrm{pr}}\right)}{\mu_{a}\left(\lambda_{\mathrm{pu}}\right)\;\Phi_{\mathrm{pu}}\left(\lambda_{\mathrm{pu}}\right)\;+\;\mu_{a}\left(\lambda_{\mathrm{pr}}\right)\;\Phi_{\mathrm{pr}}\left(\lambda_{\mathrm{pr}}\right)}$$

where $\mu_{a}$ is the known optical absorption coefficient of the protein solution, $\lambda_{\mathrm{pu}}$ and $\lambda_{\mathrm{pr}}$ are the pump and probe wavelength, and $\Phi_{\mathrm{pu}}$ and $\Phi_{\mathrm{pr}}$ are the incident pump and probe fluences. The fluences are calculated from the pump and probe pulse energies measured independently. Since the relative contribution of each pulse to the total PA signal amplitude depends on the absorption coefficient at the pump and probe wavelengths, the signals are normalized by pulse energies (or fluences) that are weighted by the absorption coefficient of the fluorophore at each wavelength as stated in equ. 1. A lowpass digital filter (Butterworth) with a 15 MHz cutoff frequency was applied to the measured signals, and the mean amplitude and standard error of repeated measurements (N = 25) were calculated.

**Fig. 3:**
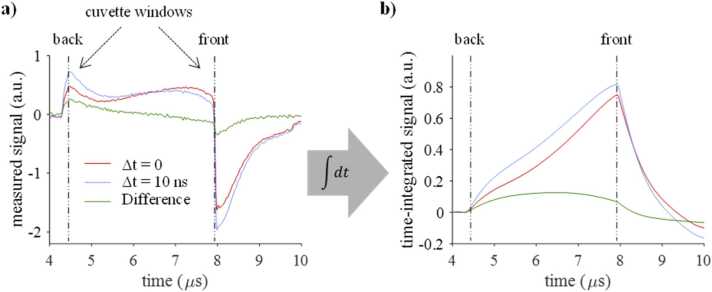
Signal processing of measured PA signals. a) Time-resolved signals measured in a solution of Katushka using simultaneous (red line) and time-delayed (blue line) pump-probe excitation ($\lambda_{\mathrm{pu}}$ = 580 nm, $\lambda_{\mathrm{pr}}$ = 650 nm) together with the difference signal (green line). b) The corresponding time-integrated signals, which provide an approximation of the axial initial pressure distributions within the cuvette.

Fig. 3a shows representative PA signals measured in a solution of Katushka. The shape of the compressive part of the signals differs from typical waveforms measured in cuvettes, as shown schematically in Fig. 1d. The difference is explained by a combination of a comparatively short cuvette pathlength and low absorption coefficients of the sample, which results in non-zero absorbed energy at the back window of the cuvette, and therefore a step change in the initial pressure. This causes an early arriving PA wave (at around 4.5 µs), which is superimposed on the later arriving acoustic wave that originates from the interior of the cuvette (5 µs < *t* < 8 µs). Similar signals are also observed in non-fluorescent absorber solutions (Supplementary Information, Figure S6).

The shape of the compressive part of the signals (Fig. 3a) is also determined by the processes involved in the thermalization of the excitation pulses during pump-probe excitation. For time-delayed probe pulses (Δ*t* = 10 ns), the absorption of the leading pump pulse leads to ground state depopulation in regions close to the optical source. This results in reduced signal amplitudes in superficial layers adjacent to the illuminated front window of the cuvette as illustrated by the blue line in Fig. 3a for 7 µs < *t* < 8 µs. Ground state depopulation also allows pump photons to propagate deeper into the sample, resulting in an increased signal at *t* ∼ 7 µs. Since most proteins have returned to the ground state by the time the probe pulse arrives, the resulting signal is equivalent to that generated by a single pump pulse (assuming negligible absorption of probe photons) [20]. By contrast, simultaneous pump and probe pulses (Δ*t* = 0 ns) cause stimulated emission, which forces the molecules from an excited state to the ground state, thus increasing the likelihood of absorption of pump photons and of multiple excitation-relaxation cycles (red line, Fig. 3b). The signal amplitude is increased at 7 µs < *t* < 8 µs, i.e., at depths near the source, and reduced at greater depths (for *t* < 7 µs), due to the overall stronger optical attenuation. From these two measurements, a difference signal is calculated (green line).

Fig. 3a illustrates that the measured PA signal amplitude is not linearly proportional to the initial pressure distribution, and hence absorbed energy. To obtain a measure of the axial PA pressure within the cuvette, a solution to the acoustic wave equation that relates pressure and velocity potential [27] was adapted (Supplementary Information, section 6). By integrating the detected signals with respect to time, a first-order approximation of the initial pressure was calculated as shown in Fig. 3b. Pump-probe spectra were obtained by plotting the time-integrated difference signal amplitude (TIDSA) as a function of the pump and probe wavelengths. The TIDSA was also plotted as a function of pump-probe time delay (for fixed pump and probe wavelengths).

Time-series data sets captured using the tomograph were corrected for pump pulse energy. 2D cross-sectional images were reconstructed using a backprojection algorithm [27]. Difference images were calculated by subtracting images acquired using simultaneous and time-delayed pump and probe pulses. A region of interest was drawn around each tube, and the maximum image intensity within each region was plotted as a function of the pump wavelength. A linear unmixing algorithm [28] was used to estimate the relative protein concentration from the spectra of the difference image intensity. The noise equivalent concentration (NEC) was calculated from the recovered concentration maps using $\mathrm{NEC}=\sigma_{c}c/\;c_{0}^{\max}\;$where c is the known concentration of the protein, $c_{0}^{\max}$ is maximum of the relative protein concentration recovered from the images, and $\sigma_{c}$ represents the background noise, i.e., the standard deviation of the relative concentrations outside the region of interest.

## 3 Results

### 3.1 Pump wavelength scans

**Fig. 4:**
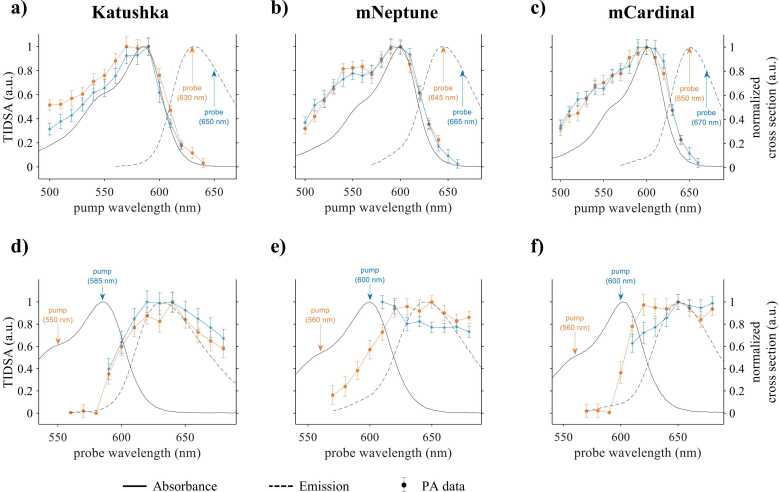
PA pump-probe spectroscopy of red fluorescent proteins. (a-c) Normalized amplitude of the time-integrated difference signal amplitude (TIDSA) measured in Katushka, mNeptune, and mCardinal as a function of pump wavelength (symbols) together with the corresponding absorption spectra (solid line) and emission spectra (dashed line). The probe wavelengths were fixed. (d-f) Normalized TIDSA as a function of the probe wavelength (symbols). The pump wavelengths were fixed as indicated. The error bars correspond to the standard error of the mean (N = 25).

Fig. 4a-c show the time-integrated difference signal measured in Katushka, mNeptune, and mCardinal (symbols) as a function of the pump wavelength for two different probe wavelengths as indicated. Normalized molar absorption spectra are shown for comparison (solid lines). The difference spectra of all proteins are in qualitative agreement with the corresponding absorption spectra, in particular between 580 nm and 650 nm. The results shown in Fig. 4a-c clearly demonstrate that the wavelength-dependence of absorption is encoded onto pump-probe difference signals. For example, the blue-shift of the difference spectrum and absorption spectrum of Katushka by 20 nm compared to those of mNeptune and mCardinal is clearly resolved.

A mismatch between the shape of pump-probe and absorption spectra is noticeable at wavelengths below 580 nm. A likely explanation is the wavelength dependence of the pulse energy of the OPO laser. The pump pulse energy is at a maximum around 450 nm and decreases with wavelength, which appears to correlate with the discrepancy between the pump-probe and absorption spectra shown in Fig. 4a-c. Although the data was normalized with respect to pulse energy, the nonlinear effect of the fluence on the difference signal could not be compensated. This was observed in all measurements irrespective of the total fluence (Fig. S2, Supplementary Information). The shape of the difference spectrum measured during a pump wavelength scan was found to be largely unaffected by the probe wavelength (Fig. 4a-c).

### 3.2 Probe wavelength scans

Fig. 4d-f show normalized spectra of the time-integrated difference signal amplitude as a function of the probe wavelength (symbols) together with the absorption spectra (solid black line) and the emission spectra (dashed black line). While the difference spectra are in reasonable agreement with the emission spectra for wavelengths greater than 620 nm, discrepancies are noticeable at wavelengths where the absorption and emission spectra overlap. If the probe wavelength falls within this range, partial absorption of probe photons increases ground state depopulation and, in turn, the difference signal amplitude. Fig. 4d-f also suggest that the probe scan spectra contain features that are specific to each protein. For example, at pump wavelengths below 560 nm (yellow line and symbols), the spectrum measured in Katushka exhibits a peak around 640 nm while those of mNeptune and mCardinal appear to plateau at wavelengths longer than 620 nm. Also, the spectrum of mNeptune increases more gradually from 570 nm to 620 nm compared to the sharper increases observed in Katushka and mCardinal. Based on these differences, it may be possible to detect fluorescent proteins based on the spectral signatures of a probe scan.

The shape of the difference signal spectra measured in mNeptune and mCardinal was found to be affected by the choice of pump wavelength. As shown in Fig. 4e and f, a pump wavelength of 560 nm produces a probe scan spectrum that is in reasonable agreement with the emission spectrum while a pump wavelength of 600 nm results in significant changes in the shape of the spectrum. The difference signal is increased in the wavelength region where absorption and emission spectra overlap compared to the plateau observed at longer probe wavelengths (*λ*_pr_ > 640 nm). Also, the observed changes in the overlap region have opposite signs with a relative increase detected in mNeptune and a decrease in mCardinal. The reason for this difference is unclear and will require further investigation. A possible explanation may lie in additional stimulated emission events by pump photons caused by the difference in the emission cross section of mNeptune and mCardinal at 600 nm and 560 nm. The magnitude of these variations was strongly affected by fluence (Fig. S3, Supplementary Information). By contrast, the difference spectrum of Katushka was found to be unaffected by the pump wavelength (Fig. 4d),which is likely due to the low stimulated emission cross section at the two pump wavelengths. It is also less affected by fluence (Fig. S3, Supplementary Information).

### 3.3 Pump-probe time delay

**Fig. 5:**
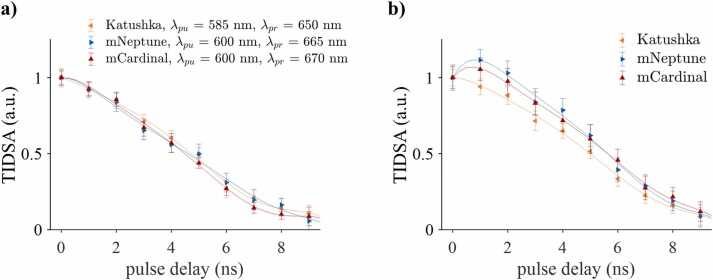
Normalized time-integrated difference signal amplitude (TIDSA) as a function of pump-probe time delay for a) pump and probe wavelengths that minimized residual absorption and stimulated emission, and b) for fixed $\lambda_{\mathrm{pu}}$ = 580 nm and $\lambda_{\mathrm{pr}}$ = 650 nm.

Fig. 5 shows the difference signal as a function of the pump-probe time delay measured in Katushka, mNeptune, and mCardinal, normalized to the signals measured at Δ*t* = 0 ns. The decay curves in Fig. 5a were measured at $\lambda_{\mathrm{pu}}$ and $\lambda_{\mathrm{pr}}$ that coincided with maximum absorption and stimulated emission cross sections whilst avoiding the region of spectral overlap. The difference signal is at a maximum for simultaneous pump and probe pulses and decreases with time-delay as the number of proteins in an excited state decreases due to spontaneous vibrational and radiative transitions. As Fig. 5a illustrates, there is no discernible difference between the decay curves. Also, the difference signal does not decrease exponentially as one may expect. This agrees with our previous studies, which showed that the convolution of the relaxation processes of fluorescent molecules, which was 1.4 ns for mNeptune and mCardinal and 2.0 ns for Katushka, with the time course of the Gaussian excitation pulse (around 4 ns) results in decay curves that are significantly longer than the fluorescence lifetime. It can also result in relatively small differences between the time delay measurements in different samples [29] as Fig. 5a illustrates. Fig. 5b shows the results of measurements with fixed pump and probe wavelengths for all proteins (*λ*_pu_ = 580 nm, *λ*_pr_ = 650 nm). Under these conditions, the difference signal at time delays between 0 ns and 4 ns exhibits changes that are specific to each protein. For example, mNeptune and mCardinal exhibit an initial increase in difference signal between 0 ns and 1 ns while that measured in Katushka decreases with time delay. These observations agree with the predictions of a rate equation model [29] and may partly be explained by residual absorption of probe photons at *λ*_pr_= 650 nm by mNeptune and mCardinal, which have the most red-shifted absorption spectra. A short time delay of 1 ns between pump and probe pulse effectively extends the time during which proteins may transition to an excited state, leading to a slight increase in excitation-relaxation cycles compared to ∆t = 0 and an increased difference signal. Katushka, by contrast, does not absorb significantly at the probe wavelength of 650 nm. The maximum excited state population, and hence difference signal, is generated for a time delay of 0 ns from which it gradually decays with increasing ∆t.

### 3.4 Fluence dependence of single-pulse and pump-probe spectra

**Fig. 6:**
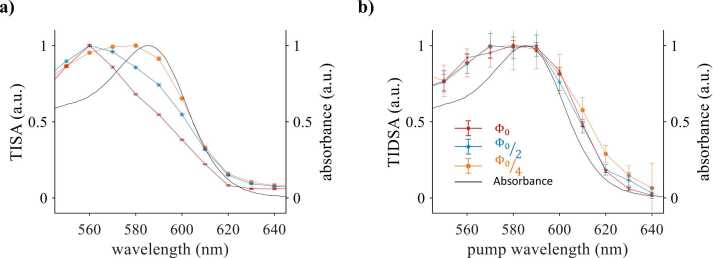
Comparison of PA spectra measured using single pulse and pump-probe excitation in a solution of Katushka at different light fluences. The maximum fluence, $\Phi_{0}$, was 12 cm. The black solid line shows the absorption spectrum for comparison. (a) The time-integrated signal amplitude (TISA) as a function of wavelength. The signals were acquired using single-pulse excitation. (b) The time-integrated difference signal amplitude (TIDSA) as a function of the pump wavelength (*λ*_pr_ = 650 nm). The error bars represent the standard error of 25 measurements.

The shape of PA spectra measured in fluorophores, including fluorescent proteins, is fluence-dependent if single-pulse excitation is used [21], [22], [23]. Fig. 6 shows normalized PA spectra measured in Katushka using single-pulse and pump-probe excitation at three fluence levels. The results for mNeptune and mCardinal are provided in Fig. S4, Supplementary Information. The results for single pulse excitation are shown in Fig. 6a, which illustrates that the spectra do not agree with the absorption spectrum. The maximum of the spectrum shifts towards shorter wavelengths with increasing fluence. The discrepancies are strongest around the absorption peak and at elevated fluences and are explained by ground state depopulation, which is caused by a combination of high photon densities and long excited state lifetimes. In addition, stimulated emission may occur at wavelengths where the absorption and emission spectra overlap. By contrast, the difference spectra in Fig. 6b show negligible fluence dependence.

### 3.5 PA tomography of fluorescent proteins using pump-probe excitation

**Fig. 7:**
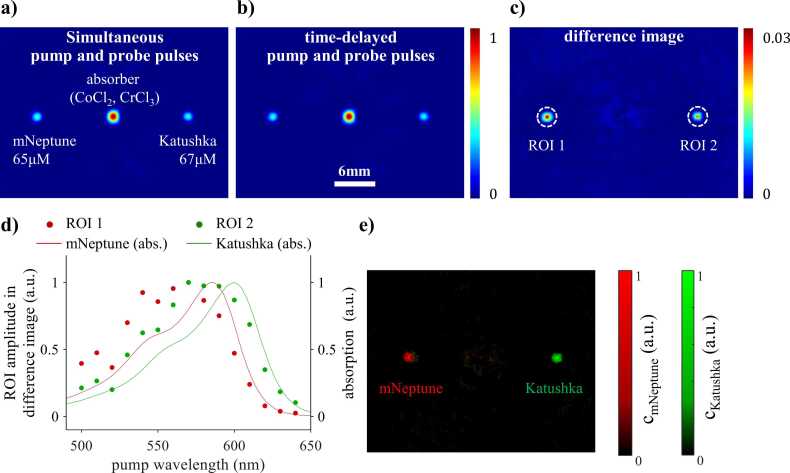
Pump-probe imaging of red fluorescent proteins in a phantom. a-b) Reconstructed cross-sectional image of tubes filled with mNeptune, Katushka, and a nonfluorescent absorber (solution of CoCl_2_ and CrCl_3_) acquired at *λ*_pu_ = 590 nm, *λ*_pr_ = 650 nm, and Δ*t* of 0 ns and 10 ns. c) Difference image calculated from a) and b). The dashed circles mark regions of interest that correspond to the location of the tubes filled with Katushka and mNeptune. d) Normalized peak difference image intensity of the regions of interest in c), as a function of the pump wavelength (symbols). Normalized absorption spectra of the proteins are shown for comparison (solid lines). e) The relative concentrations of mNeptune (red) and Katushka (green) determined from multiwavelength difference images using linear unmixing.

Image data sets of a tissue phantom containing three tubes filled with solutions of Katushka, mNeptune, and a nonfluorescent absorber were acquired using a pump wavelength scan. Fig. 7a-b show the cross-sectional images acquired with simultaneous pulses (Δ*t* = 0 ns) and time-delayed pulses (Δ*t* = 10 ns). Fig. 7c shows the corresponding difference image in which the location of the tubes filled with Katushka and mNeptune solutions is visualized with high image contrast while the contribution of the non-fluorescent absorber is removed. Fig. 7d shows the maximum difference image intensity (symbols) within the regions of interest indicated by dashed circles in Fig. 7c as a function of pump wavelength. The spectra correlate with the absorption spectra of the proteins (solid lines). Fig. 7e shows maps of the relative concentration of the proteins recovered from difference images acquired at wavelengths ranging from 600 nm to 640 nm using linear inversion. The location and relative concentrations of Katushka (green) and mNeptune (red) within the tissue phantom can be discerned clearly from Fig. 7e. The NEC of mNeptune and Katushka achieved in this experiment is estimated as 0.50 µM and 0.62 µM, respectively.

## 4 Discussion

The results presented in Section 3 demonstrate that pump-probe excitation can be used to recover information on the photophysical properties of fluorescent contrast agents from difference signals acquired whilst scanning the pump wavelength, the probe wavelength, and the time delay. The difference signal spectrum measured during a pump wavelength scan was found to agree with the reference absorption spectrum of the protein solution. The key advantage of this approach lies in its spectral specificity, which allows maps of the relative concentration of two fluorescent proteins to be recovered from difference images using the reference absorption spectra of the proteins and simple linear unmixing. Specificity and sensitivity were maximized when the inversion was applied to images acquired at pump wavelengths between 600 nm and 640 nm rather than the full image data set (Fig. 7e). This may be explained by the difference absorption spectrum of Katushka and mNeptune, which is greatest in this wavelength region (Figure S5, Supplementary Information). The results show that multiplexed imaging of fluorescent proteins or dyes is feasible using this approach. In addition, it may be possible to detect changes in environmental parameters, such as pH and ion concentrations, based on changes in the absorption spectrum of fluorophores [30], [31]. It should be borne in mind, however, that difference spectra measured *in vivo* are likely to be affected by spectral coloring, which may reduce sensitivity and specificity. To minimize this effect, difference images should be acquired over a narrow wavelength range. NEC values of hundreds of nM were estimated from the concentration maps shown in Fig. 7e. Given that reported intracellular concentrations of fluorescent proteins range from nM to several µM [32], [33], [34], [35], the NEC indicates that the sensitivity of the method is sufficiently high for *in vivo* PA reporter gene imaging. Given the typical range of optical attenuation in tissue [36], the NEC values estimated for Katushka and mNeptune would correspond to a maximum imaging depth of 9 mm to 11 mm. Another advantage of pump wavelength scanning is the robust and fluence-independent recovery of difference signal spectra (Fig. 6b). The discrepancies between measured difference spectra and absorption spectra are probably caused by the wavelength dependence of the pulse energy provided by the OPO laser. While the mismatch between measured PA spectra and literature absorption spectra is primarily caused by long excited state lifetimes, the underlying mechanism during pump-probe excitation is different to that observed for single pulses (Fig. 6a). The amplitude of the difference signal is dependent on the ratio of the density of proteins and the density of (pump) photons. At high photon densities (or fluences), more proteins per volume are transferred to an excited state, causing ground state depopulation. During conventional single-pulse excitation, an increase in fluence reduces signal amplitude (Fig. 6a). During pump-probe excitation, by contrast, an increase in the pump fluence causes stronger ground state depopulation, which in turn results in an increase in the difference signal as shown in Fig. 4a-c and Fig. 6b for wavelengths below 580 nm. This effect can be minimized by normalizing the OPO output using, for example, variable neutral density filters [23]. The robustness of the pump-probe data makes this approach particularly suited to the detection of small changes in the optical properties due a stimulus, such as pH. By contrast, PA spectra acquired using single pulse excitation were shown to be strongly affected by fluence (Fig. 6a), severely limiting the validity of linear unmixing algorithms that are reliant on constant optical properties.

Difference spectra acquired during a probe wavelength scan also showed characteristic features for each fluorescent protein. However, reasonable qualitative agreement between difference and reference emission spectra was only observed at comparatively low fluences and for pump and probe wavelengths that avoided the spectral region where absorption and emission spectra overlap. At high fluences and for excitation wavelengths within the overlap region, the shape of the difference spectra changed significantly. The cause of these discrepancies may be explained by nonlinear processes during the thermalization of the optical energy due to residual absorption of the probe pulse and stimulated emission by the pump pulse. Although the interpretation of these phenomena requires further study, the results nevertheless show that difference spectra contain information on the wavelength dependence of the stimulated emission cross section. This method therefore also has the potential to be used for the multiplexed detection of fluorophores, including fluorescent proteins, and monitoring of biochemical parameters in the microenvironment via their effect on absorption and emission spectra [30], [37].

Varying the time delay between pump and probe pulses produced insignificant differences between the measurements made in each protein when the excitation wavelengths did not coincide with the spectral region where absorption and emission of mNeptune and mCardinal overlap (Fig. 5a). Interestingly, for pump and probe wavelengths within the spectral region where absorption and emission of mNeptune and mCardinal overlap, the difference signals showed a dependence on time delay that was specific to each protein (Fig. 5b). This was particularly noticeable for small time delays and may be explained by additional contributions due to residual absorption and stimulated emission events. It may therefore be advantageous to exploit these phenomena by deliberately designing fluorescent probes in which this type of optical crosstalk is maximized (at a fixed time delay). By harnessing a combination of differences in lifetime, and the absorption and emission cross sections, the detection of multiple fluorescent probes and their interrogation to measure changes in the microenvironment using time delay imaging [38] may be more sensitive compared to relying on the detection of differences in lifetime alone.

The methods described in this paper may allow the use of fluorophores, such as genetically expressed fluorescent proteins, as biosensors by enabling the detection of changes in absorption and emission spectra, and excited state lifetime, in response to an environmental stimulus, such as pH [37], [38], [39] and monitoring cellular senescence based on changes in intracellular pH and metabolic activity [40]. Other applications include imaging of protein crowding [32], the visualization of ATP concentration [41] to study its role in apoptosis and necrosis [42], and cellular processes, such as interactions of proteins with ions [43], [44] and ligands [45].

## 5 Conclusions

Three methods for PA spectroscopy and imaging of fluorophores based on pump-probe excitation have been investigated. The ability to generate difference images that selectively show the contribution from fluorophores while suppressing non-fluorescent absorbers, i.e., most endogenous tissue chromophores, makes this technique highly advantageous for molecular imaging applications. By scanning the pump and probe wavelengths, difference spectra were obtained that correlate with the characteristic absorption and emission spectra of the proteins. By measuring the difference signal as a function of the time delay between the pump and probe pulses, decay curves were obtained that exhibit characteristic differences between the proteins investigated in this work. Difference spectra were found to be less affected by fluence compared to spectra obtained using single pulse excitation, which has the potential to improve sensitivity and specificity when compared to conventional multiwavelength imaging and linear unmixing approaches. This study has demonstrated that pump-probe imaging in combination with scanning the pump wavelength, probe wavelength, and time delay may allow multiplexed imaging of multiple fluorescent proteins.

## Funding

This work was supported by the European Regional Development Fund for Saxony-Anhalt (Grant no. EFRE: ZS/2016/04/78115), 10.13039/501100001659Deutsche Forschungsgemeinschaft, and Land Sachsen-Anhalt (project no. LA 3273/8-1, INST271/385-1), and core funding of Martin-Luther-Universität Halle-Wittenberg.

## CRediT authorship contribution statement

**Farzin Ghane Golmohamadi:** Writing – review & editing, Writing – original draft, Visualization, Validation, Software, Methodology, Investigation, Formal analysis, Data curation. **Amna Mehmood:** Writing – review & editing, Validation, Methodology, Investigation, Formal analysis, Data curation. **Hoang Trong Phan:** Writing – review & editing, Writing – original draft, Validation, Methodology, Investigation, Formal analysis, Data curation. **Franz-Josef Schmitt:** Writing – review & editing, Validation, Resources, Methodology, Investigation, Formal analysis, Data curation. **Jan Laufer:** Writing – review & editing, Writing – original draft, Visualization, Validation, Supervision, Software, Resources, Project administration, Methodology, Investigation, Funding acquisition, Formal analysis, Data curation, Conceptualization.

## Declaration of Competing Interest

The authors declare that they have no known competing financial interests or personal relationships that could have appeared to influence the work reported in this paper.

## Data Availability

Data will be made available on request.
